# A Memoir on Extra-Uterine Gestation

**Published:** 1840-07

**Authors:** 


Art. VII.—*A Memoir on Extra-Uterine Gestation.* By Dr. W. Campbell, of Queen's College, Edinburgh, &c. &c.—*London,* 1840. 8vo, pp. 154.

This is a most elaborate memoir, and very creditable to the industry of the author; sometimes, indeed, the feeling crossed our minds during the perusal of it that he had been a little too painstaking; for doctrines, of which he believes little or nothing, and cases to which he attaches an equal degree of credit, are detailed and commented on with as much care as if they were valuable additions to our stock of medical learning. We do not perceive that Dr. Campbell has succeeded in throwing any new light upon the subject of extra-uterine gestation, and, to deal justly with him, we scarcely expected it. They, however, who are anxious upon this subject, and desire to peruse an accumulation of cases from the earliest periods to the present moment, may consult this little volume with more advantage than any of the essays upon it which are contained in various other works.

